# Interaction of β-Sheet Folds with a Gold Surface

**DOI:** 10.1371/journal.pone.0020925

**Published:** 2011-06-07

**Authors:** Martin Hoefling, Susanna Monti, Stefano Corni, Kay Eberhard Gottschalk

**Affiliations:** 1 Theoretical and Computational Biophysics, Max Planck Institute for Biophysical Chemistry, Göttingen, Germany; 2 CNR Istituto di Chimica dei Composti OrganoMetallici (ICCOM), Pisa, Italy; 3 Centro S3, CNR Istituto Nanoscienze, Modena, Italy; 4 ZIK HIKE Centre for Humoral Immune Reactions in Cardiovascular Disease, Universität Greifswald, Greifswald, Germany; King's College London, United Kingdom

## Abstract

The adsorption of proteins on inorganic surfaces is of fundamental biological importance. Further, biomedical and nanotechnological applications increasingly use interfaces between inorganic material and polypeptides. Yet, the underlying adsorption mechanism of polypeptides on surfaces is not well understood and experimentally difficult to analyze. Therefore, we investigate here the interactions of polypeptides with a gold(111) surface using computational molecular dynamics (MD) simulations with a polarizable gold model in explicit water. Our focus in this paper is the investigation of the interaction of polypeptides with β-sheet folds. First, we concentrate on a β-sheet forming model peptide. Second, we investigate the interactions of two domains with high β-sheet content of the biologically important extracellular matrix protein fibronectin (FN). We find that adsorption occurs in a stepwise mechanism both for the model peptide and the protein. The positively charged amino acid *Arg* facilitates the initial contact formation between protein and gold surface. Our results suggest that an effective gold-binding surface patch is overall uncharged, but contains *Arg* for contact initiation. The polypeptides do not unfold on the gold surface within the simulation time. However, for the two FN domains, the relative domain-domain orientation changes. The observation of a very fast and strong adsorption indicates that in a biological matrix, no bare gold surfaces will be present. Hence, the bioactivity of gold surfaces (like bare gold nanoparticles) will critically depend on the history of particle administration and the proteins present during initial contact between gold and biological material. Further, gold particles may act as seeds for protein aggregation. Structural re-organization and protein aggregation are potentially of immunological importance.

## Introduction

The interaction of inorganic surfaces with biomolecules like peptides and proteins is central to biotechnology [1], being involved, e.g., in biosensors, biomaterials [2] or the biological use of nanoparticles [3]. Further, aspects of nanotoxicity of inorganic particles may be based on particle-protein interactions [4], [5]. Despite such importance, the microscopic understanding of how proteins interact with inorganic surfaces is still limited. Besides relatively well-characterized bonds such as *Cys* on gold [6], little is known about the exact nature of the interaction. However, the interaction between proteins and inorganic surfaces is based on well-defined principles as demonstrated by the ability to genetically engineer proteins specific for and discriminating between given surfaces [7]. Yet, technical limitations hamper the detailed structural and dynamical experimental characterization of biomolecular interactions with surfaces [8]. Computational methods, on the other hand, are well-suited to atomistically describe the effect of surfaces on biomolecules [9], [10], [11]. One important biocompatible metal widely used in biotechnological application is gold [12]. Being a metal, gold polarization effects are important and realistic simulations need to include them. Only recently, force fields suitable for this task became available [13]. Using such a force field in earlier work, we analyzed the adsorption of single amino acids (the 20 natural ones) onto gold surfaces [14], [15], [16]. However, the complex cooperative behavior of a polypeptide [17], [18], [19], [20], [21], [22] may significantly alter the adsorption properties. Therefore, our focus in this work is to analyze the association of a complex biomolecule with a gold(111) surface. Our earlier results indicated that amino acids with an intrinsic propensity to form β-sheets are predisposed to interact with gold surfaces [15]. We therefore concentrate here on simulating the adsorption of two polypeptides with high β-sheet content using a polarizable gold(111) surface as parameterized in the force field GolP [13]. First, we simulated by molecular dynamics an oligopeptide (RAD16II) forming a fibrillious structure. It is a spontaneous self-assembling amphiphilic peptide, known to form hydrogel-like matrices that support cell attachment, proliferation and differentiation [23], [24]. The structure of the elastic properties of filaments formed by RAD16II have been investigated by atomistic simulations [25]. This peptide has been suggested as a coating to make inorganic surfaces biocompatible, and the interaction of RAD16II fibers with surfaces other than gold (TiO_2_) has been studied by classical MD simulations [24]. These studies showed that the fibrous structure is maintained in typical MD simulation times Fibrillious structures are important for many protein-folding related diseases like Alzheimer or bovine spongiforme encephalopathy (BSE). The peptide absorbs quickly in our simulations, is stable on the gold surface and shows no tendency of unfolding. For this peptide, which is rich in *Arg*, we characterize how the *Arg* side chain can penetrate the water easily, creating a stable initial anchoring points.

Of particular importance, in terms of nanotechnological applications, is the interaction of a gold surface with members of the extracellular matrix (ECM). Not only are ECM proteins used as anchors for cells on gold electrodes, these proteins will also be the first encountered by gold nanoparticles administered to organisms [26], [27]. One of the most important members of the ECM proteins is Fibronectin. This protein is involved in cell migration, adhesion, and metastasis [28]. The large size of Fibronectin and the lack of a full-length atomistic structure of this protein prevent the simulation of the adsorption of the whole protein on the gold surface. However, a crucial part of this protein is composed of the FibronectinIII modules 9 and 10 (FNIII9–10). FNIII9–10 contains the RGD binding site in FNIII10 as well as a synergy site in FNIII9, important for integrin-mediated cell adhesion and force-initiated signalling [29]. The structure of the two domains is known and the modest size of this fragment allows atomistic simulations in a reasonable time frame. Due to the biological importance of FNIII9–10, appreciating its interaction with gold not only fosters our basic understanding of biomolecule-metal interactions in general, but has technological and health-care implications. Therefore, we report here long atomistic molecular dynamics simulations of the type III fibronectin domains 9 and 10 (FNIII9–10) on the polarizable gold(111) surface in explicit water.

We show that gold strongly interacts with FNIII9–10, but does not lead to unfolding of this β-sheet rich protein within the simulation time. Also in this case, *Arg* plays an important role for penetrating the dense water layer covering the hydrophilic gold surface. In *in silico* mutational studies, we show that an *Arg*→*Lys* mutation does not alter the general adsorption pattern, while an *Arg*→*Val* significantly changes the proteins behavior, underlining the importance of charges for the adsorption process. Our results have implications for assessing possible health risks associated with gold particles and may serve as a starting point for the design of gold binding proteins.

## Results and Discussion

### Model peptide

First, we have performed a simulation of a β-sheet like structure formed by the oligopeptide RAD16II. This is a 16-mer peptide with a RARADADARARADADA sequence that spontaneously self-assembles in anti-parallel β-sheet fibers [30], [31]. Hence, it is a relevant model system for other β-sheet forming peptides like the Alzheimer precursor protein. Further, RAD16II represents an ideal system to study the basics of β-sheet forming peptide adsorption. With *Arg* and *Asp* in proximity, it offers two competing interaction partners with opposite charge. However, it is overall uncharged. Its rigid, planar structure facilitates the analysis, in particular in comparison to the complex surface topology of a multi-domain protein. With its β-sheet structure, it is also a good simplified representation of the β-sheet fold of Fibronectin.

**Figure 1:**
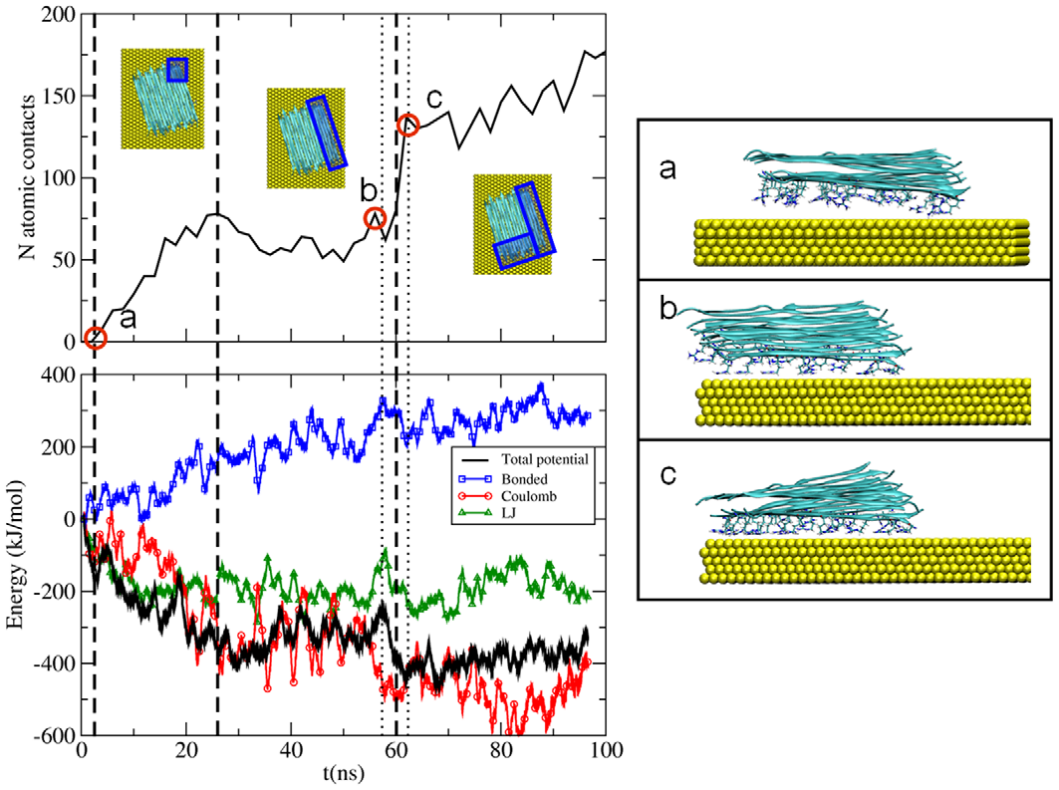
Evolution of the atomic contact number between the fiber and the Au surface, and of the corresponding adsorption energy. In the right panels, relevant snapshots from the dynamics are shown. The blue box in the top view indicates the adsorbed parts with progressing simulation time.

The *Arg*-rich β-sheet quickly replaces water molecules, thus forming strong peptide-surface contacts. However, water replacement and peptide adsorption are not a continuous process despite the symmetric and regular structure of the peptide. The adsorption process can be divided into four different phases (Fig. 1). The first contact is formed after ∼3 ns. Then, a gradual increase in the number of contacts takes place during the subsequent 20 ns leading to the adsorption of all the *Arg* residues of the lateral oligopeptide molecule. After a ∼35 ns plateau, the sharp increase in the number of contacts in the interval 58–60 ns corresponds to the adhesion of the edge of the β-sheet perpendicular to the adsorbed portion of the molecule (Fig. 1B and 1C). The sharpness of the transition indicates that the whole edge is cooperatively getting into contact with the surface. Cooperativity is enforced by the H-bond network between the chains, which fosters a zipping-like mechanism. After this jump, the number of contacts gradually increases till the end of the simulation. No sign of structural changes in the β-sheet is seen during the dynamics.

The evolution of the adsorption energy for the peptides approaching the surface is also shown in Fig. 1, so as to be compared with the number of atomic contacts. The various energy components (bonding, electrostatics and Lennard Jones, LJ) are depicted as well. Since the contact of the peptide with the surface is steadily increasing with time (besides local fluctuations), the energy curve is representative of the adsorption energy landscape of the peptide while it settles on the surface, neglecting entropic contributions. The profile of the adsorption energy, although fluctuacting, is clearly correlated with the number of atomic contacts. The increase in the number of contacts during the 0∼25 ns time interval is accompanied by a steady decrease in the adsorption energy. This was initially due to the Lennard Jones term (∼20 ns) and then also to electrostatic interactions. From 25 to 58 ns (i.e., the dotted line) the number of contacts has a plateau and the potential energy is almost constant. Just before the number of contacts ramps up (i.e., before the dotted line at 58 ns), there is a decrease in the electrostatic energy and an increase in both bonding and LJ terms, suggesting that during this period the system behavior is guided by the electrostatic interactions, which temporarily induce the formation of an higher-energy structure (the total potential energy has a positive fluctuation). However, this conformation rapidly relaxes (region between the dotted lines) via a rearrangement of the sidechains that corresponds to the sharp increase in the number of atomic contacts described above. After this step, further stabilization of the system configuration is achieved through a steady increase in the atomic contacts which is clearly evidenced after point c. Notably, the bonding term (blue) is increasing throughout the dynamics, in order to maximize the interaction with the surface. Indeed, the internal geometry of the peptide gradually departs from the optimal conformation adopted by the isolated molecules.

**Figure 2:**
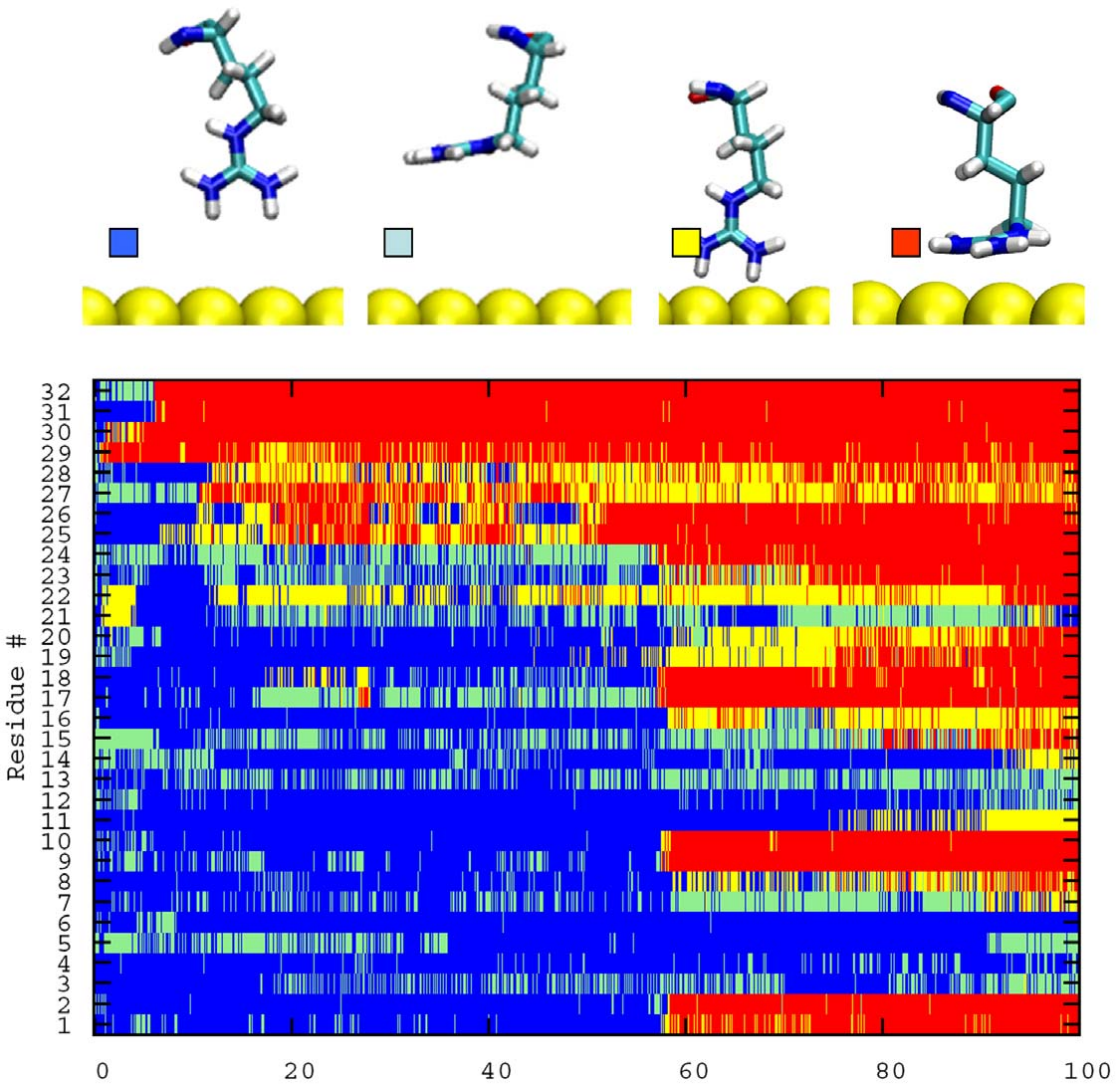
Contact and orientation map for the 32 gold-exposed *Arg* of the RAD16II peptide. The color code (defined in the upper panels) indicates whether *Arg* is in contact with the surface or not, and whether its side chain orientation is perpendicular or parallel to the surface. *Arg* is defined to be in contact when any of its atoms is within 0.35 nm of the surface. Side chain is defined to be parallel to the surface when all the guanidinium C-N bonds have angles with the surface smaller than 30°, otherwise orientation is called perpendicular. If a looser definition of parallel orientation is used (angles smaller than 45° instead of 30°), the number of first contacts taking place in the perpendicular orientation is still >70% of the total.

**Figure 3:**
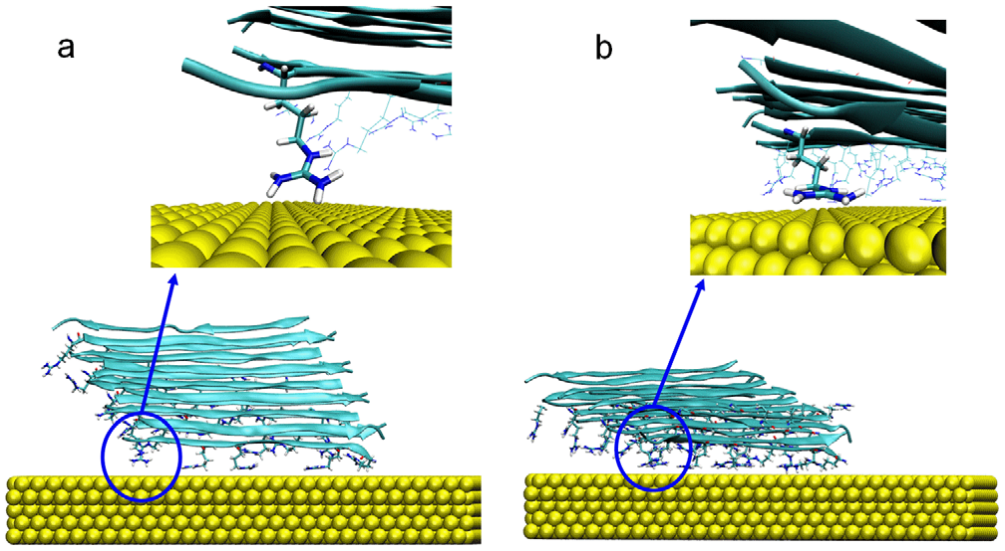
Switching of *Arg* side-chain conformation during the association process. *Arg* contacts the surface having the plane of the side chain perpendicular to it (Fig. 3a). After a few ns, the plane of the methylguanidinum moiety has changed its orientation becoming parallel to the surface.

Strikingly, the interactions of *Arg* side chains with the gold layer are the driving force of peptide-surface the association. The electrostatic energy minimum that accompanies the sharp increase of atomic contacts at 58–62 ns corresponds to several *Arg* finding their way to the surface (Fig. 2). In all the described adsorption steps, the *Arg* capability to penetrate the water layer is fundamental, forming the initial peptide-gold contact. In >80% of first contact events, the *Arg* side chain is perpendicular to the surface (Fig. 2), minimizing the number of water molecules to be displaced (Fig. 3). Afterwards, the side chain changes its conformation becoming parallel to the gold surface. Such an adsorption geometry has already been observed for an isolated *Arg*, competing with a second geometry with an adsorbed backbone. The observed orientation is the preferred orientation found for methylguanidinium (i.e., the molecule representing the *Arg* side-chain) in water [16]. Our simulations strongly indicate that *Arg* may play a dominant role in the adsorption of polypeptides.

### Fibronectin Close to Surface

**Figure 4:**
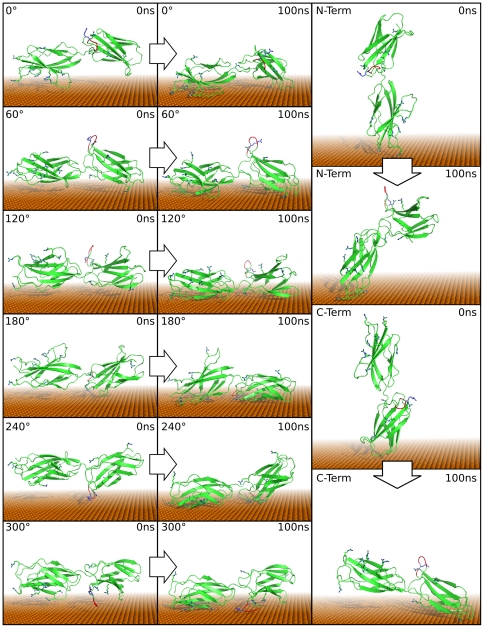
Starting and final conformations of FN. On the left side, the six rotations of the conformation parallel to the surface are shown while the right side depicts the perpendicular conformations with N- or C-Terminus close to the surface. The integrin-binding RGD-loop is indicated in red. Domain 9&10 from 1FNF were used for the simulations. First the principal axis was aligned parallel to the surface. Then, stepwise rotations of 60° around the principal axis produced the initial conformations. The obtained structures were separated by one water layer from the surface.

**Figure 5:**
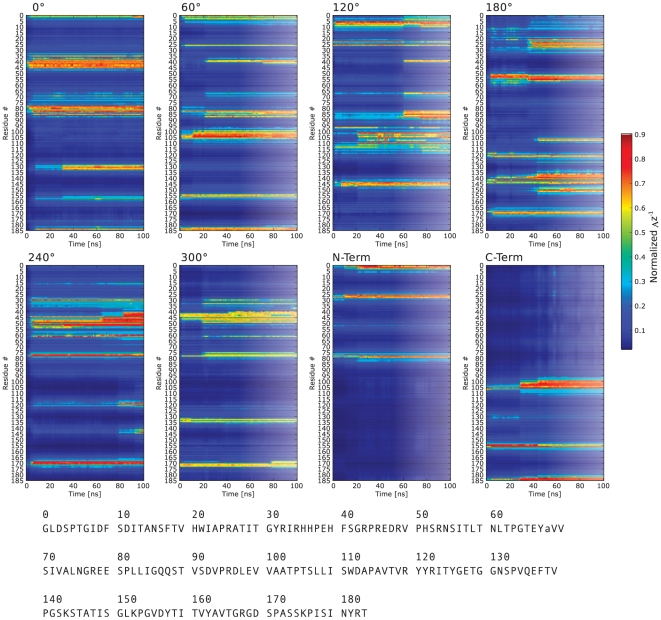
Time resolved adsorption of FN residues. The distance from closest atom of each residue from the surface is shown during each 100 ns simulation color coded according to Δz^−1^ where 1.0 corresponds to the minimum distance and 0 to ∞. Residue # refers to the protein sequence reported below.

As the next level of complexity, we simulated the last steps of adsorption of FNIII9–10. To this end, we placed the protein in eight different orientations over the gold surface with approximately one water layer between FN9–10 and the metal surface (Fig. 4) and monitored the protein-gold distances over a simulation of 100 ns per orientation. With these simulations, we save computational time due to the reduced need to simulate the diffusional encounter of the protein with the surface. This allows us to sample many different protein-gold orientations. Effects of diffusion are considered later in this work. Well-defined contact areas are formed quickly (Fig. 5), being stable over the whole length of the simulation. Within the 100 ns simulation, no significant re-arrangement of the initial adsorption geometry occurs. The contacts between the protein and the metal surface do not break once formed (Fig. 5). This demonstrates the strong interaction of the protein with the surface. In a variety of Atomic Force Microscopy experiments, it has been demonstrated that adsorption of proteins on surface leads to interactions that are so strong that the individual protein domains unfold rather than detach from the surface when force is applied, in line with our results here [32], [33]. Our results furthermore indicate that once adsorbed, the proteins are in a kinetically trapped state (translational and rotational), which prevents the reorientation to a potentially more optimized adsorption geometry. However, although our simulations of altogether more than one µs approach experimental time frames, slow re-orientation processes on longer timescales may occur [34], [35].

**Figure 6:**
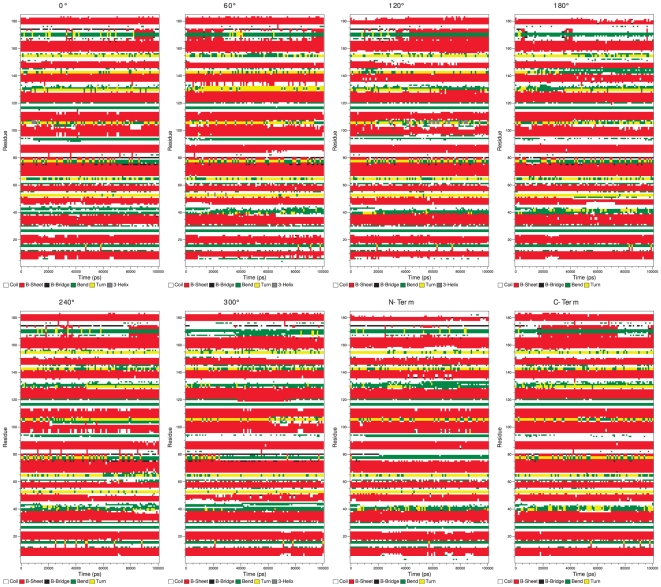
Secondary structures changes during simulation time. No significant conversions could be observed. The secondary structure maps were created with do_dssp, the Gromacs interface to dssp.

**Figure 7:**
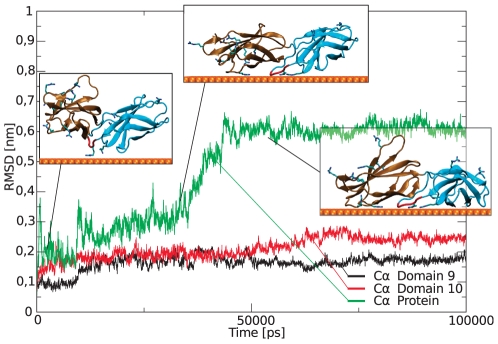
Cα RMSD of the two domains and the entire protein. The first inlet shows the initial adsorption while the others depict the twisting of the domains in the region between 35 ns and 44 ns. *Arg* are shown in licorice representation. See Fig. 8 for all simulations.

**Figure 8:**
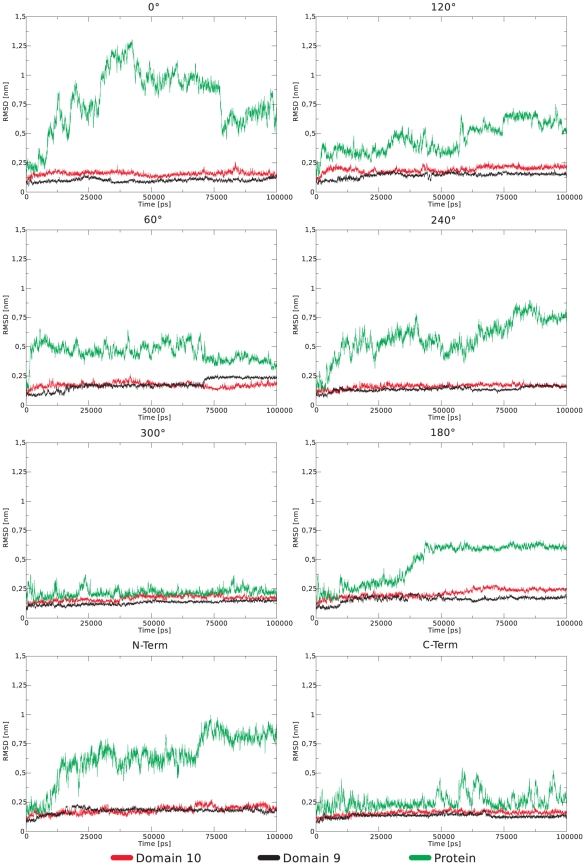
RMSD of Domain 9 & 10 and the entire protein from all simulations. Only Cα - atoms are considered. Major changes are observable only in the RMSD of the entire protein.

Despite the strong interaction with the gold surface, the protein does not unfold on the gold surface within the simulation time in either of the simulations. The β-sheet content is virtually unchanged (Fig. 6 [36]), and no major conformational changes within each domain occur. The Cα RMSD of the individual domains remains below 3 Å (Fig. 7 and 8). However, the total root mean square deviation (RMSD) of the two domains together shows drastic changes, indicative of the gold-induced remodeling of the domain-domain orientation. The stepwise increase in the total RMSD indicates collective structural transitions between different energy minima, and the resulting relative domain orientation is rigid.

Although the individual domains remain rather stable, the protein adapts to the gold surface. The originally roundish surface of the protein becomes planar at the site of adsorption, constituting something like an induced-fit mechanism.

Like the model peptide, FNIII9–10 has a very high β-sheet content. Based on simulations of individual amino acids on gold surfaces [15], we proposed earlier that gold surface might in fact stabilize β-sheets, in agreement with the stable individual domain folds observed here.

The behavior of α-helical domains on gold may be different and will be the focus of future studies.

### Inclusion of Diffusion

As a next step, we were also interested in the diffusional states of FN further away from the protein. To investigate this, we placed FNIII(9–10) between two gold surfaces with a separation of 8 Å to one of the gold surfaces resulting in a distance of 23 Å to the other gold surface. This allows the protein to freely choose on which side to adsorb and to change the relative orientation of the protein relative to the gold surface during the diffusional phase. We simulated the protein starting from identical conformations but from two different sets of randomly Maxwell distributed atomic velocities. Due to the chaotic behavior of many particle systems, simulation trajectories with different initial atomic velocities quickly diverge in the sub nanosecond regime.

### Fast Adsorption

In both cases, FNIII(9–10) quickly adsorbed on the gold surface. However, despite the identical starting conformation, the association times and geometries vary significantly, demonstrating that the protein is still in a diffusional state at the start of the simulation. This behavior is consistent with free energy profiles calculated for the adsorption of single amino acids to the gold surface. There, strong attraction was only observed at distances below 8 Å [15], [16].

The fast association reaches the diffusional limit. From simulations in water, we found that the 3D diffusion constant is D = 0.1031 (+/− 0.0215) 1e-5 cm∧2/s. This translates to a mean travelled length of L = 0.786 nm within 1 ns. In the simulations with a separation of 0.8 nm from the gold surface, the first contact times were 0.4 ns and 21 ns. Thus, in one of the two simulations, the adsorption time is in fact faster than expected for random diffusion. It indicates that no major energy barriers exist on the adsorption trajectory. Earlier results on the adsorption of **individual** amino acids support that the adsorption of biomolecules on gold surfaces is energetically predominantly a downhill process with only a small dewetting barrier. The comparable behavior of the complex biomolecule shows that no energetically costly conformational changes need to occur for the adsorption. The fast adsorption is further indicative of a rather unspecific binding interface. Once adsorbed, no desorption is observed within our simulation time. Further, also in this case we observe no major rearrangements of the relative orientation of the gold and the protein once adsorbed.

### Physicochemical Properties of the Protein Patch Forming the Initial Contact

**Figure 9:**
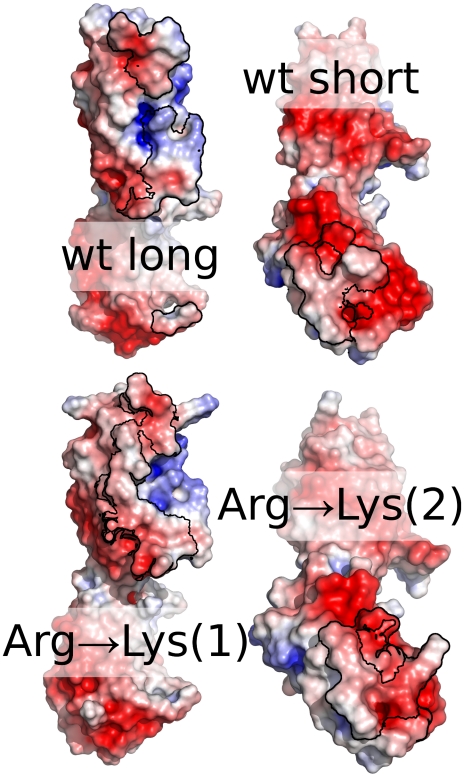
Electrostatic potential on the initial adsorption sites. The electrostatic surface potential on the solvent accessible surface is mapped on the van-der-Waals surface of FN. The first contact region is surrounded by a black line. Most parts of the contact regions exhibit a low electrostatic surface potential.

After formation of a first stable contact, the protein does not diffuse away from the gold surface. Hence, the initial contact between protein and surface is the determining step in the adsorption process. Understanding the physicochemical properties of this patch may allow identifying putative gold binding sites in other proteins without reverting to time-consuming atomistic MD simulations. Two different surface patches form this initial contact in our simulations. This underlines the rather unspecific nature of the adsorption process. To analyze the physicochemical properties of the contacting surface patches, we mapped the electrostatic potential of proteins as calculated with an adaptive Poisson-Boltzman approach [37] on the solvent accessible surface. Patches with electrostatic potential close to zero, surrounded by area with potential values deviating from zero, form this initial contact (Fig. 9). Earlier results on the free energy of adsorption of individual amino acids showed a different trend: there, positively charged amino acids formed stronger interactions than apolar amino acids [15]. Different reasons may contribute to reconcile these apparently contradicting findings. The first and more fundamental is that the adsorption free energy of the amino acid is a thermodynamic quantity, while the present results for the entire proteins refer to the dynamic behavior (i.e., to the kinetics) of the system. To bring a protein patch in contact with the surface, it is necessary to desolvate such patch. Therefore, a dewetting barrier should be overcome. Such a barrier, which can be seen even for single amino acids [15], will be larger for protein patches that bound water more tightly, e.g, patches at potentials far from zero. As a result, patches with a potantial far from zero will dewett more slowly. Hence, the here observed first contacts may be determined by the low dewetting barrier of patches with a potential close to zero.

Of course similar effects related to desolvation may modify the adsorption thermodynamics for the whole protein, not just the kinetics. In fact, in the earlier thermodynamics studies on single amino acids, the charged side chain in proximity to the surface is still exposed to solvent. Hence, no major desolvation penalty is paid for individual charged amino acids, while simultaneously attractive interactions between the amino acid charge and the polarizable gold surface are possible. In our studies of a whole protein reported here, the sidechains are shielded from the solvent by the neighboring amino acids once adsorbed. This shielding effect may tip the balance so that the interactions between polarizable gold surface and the charged amino acids together with the entropic gain obtained from freeing the water molecules bound to the gold surface and the charged surface patches cannot compensate for the loss of solvation enthalpy.

The physicochemical properties observed here resemble the architecture of protein-protein interaction sites. However, protein-protein interactions are highly specific and the results of long evolutionary process, while the here observed interactions are not physiological and unspecific. Hence, the interactions observed here can be regarded as a model system for protein-protein interactions before evolutionary optimization. This supports the earlier notion that protein-protein interaction sites evolved from unspecific interactions, formed by patches with an intrinsic propensity to aggregate. These unspecific interaction sites were then optimized by mechanisms inducing shape and charge complementarity. This is facilitated by the plasticity of both interaction partners and the complexity of a protein surface, allowing for a high variety in shapes. The challenge for designing specific protein-to-surface interaction lies in the fact that the surface is flat, isotropic and not adaptive, so that only one interaction partner is available for evolutionary optimization.

### Adsorption is a step-wise process

Analyzing the contact times of individual amino acids of the protein with the gold surface shows a step-wise process. First, a contact is initiated by a few amino acids. This is then followed by a very fast cooperative adsorption of a complete surface patch. Despite the more complex nature of the protein, this adsorption sequence is very similar to the adsorption of the model peptide. This suggests that the basic features observed in the model peptide are well preserved in complex systems. The surface patches of FNIII9–10 behave like independent binding units. This hints at a modular architecture of the binding interface. Modular architectures of binding interfaces have previously been proposed for protein protein interactions, demonstrating a conceptual similarity. This raises the question whether a certain modularity is an intrinsic property of interactions involving proteins, stemming from the self-organization of the polypeptide chain during folding.

Also here *Arg* seems to be overrepresented in the early initiation phase of the interaction, underlining the particular ability of *Arg* to penetrate the final water layer, establishing a first contact and facilitating the dewetting transition.

### Binding-Site Reorientation

**Figure 10:**
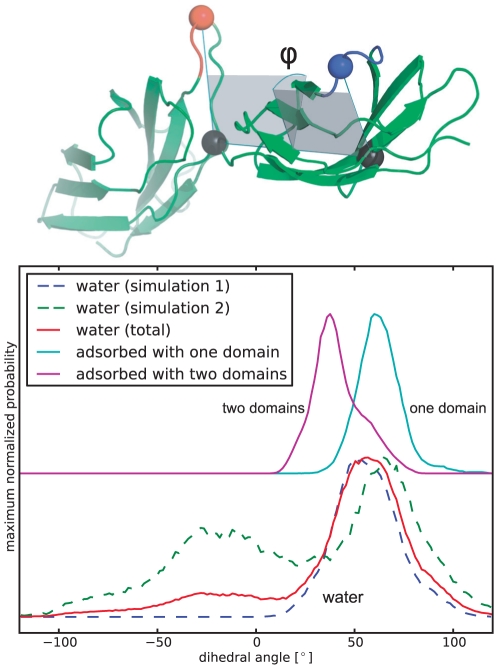
RGD and synergy site orientation during adsorption. Dihedral angle between the RGD (blue), two representative residues (black) and the synergy site (red) in FN is shown top. Below the angular distribution of this dihedral is shown for two simulations of FN in water and for two adsorption simulations. The individual water angle distributions are shown as dashed lines, the cumulative distribution as solid line. A narrowing of the distribution is observed after adsorption. Further, for the simulation with both domains adsorbed, an additional shift in the distribution is seen.

Binding of cells to fibronectin induces cellular signaling cascades. It has been shown that two binding sites in FNIII(9–10) are of particular importance for cellular force sensing: the main RGD binding site located in FNIII(10) and the synergy-site located in FNIII(9). Since biological recognition processes rely on the fine-tuned relative orientation of different binding partners, it is expected that changes in the relative orientation impact binding and signaling properties. Therefore, we analyzed the relative orientation of the synergy site and the main RGD binding loop as indicated by the dihedral angle distribution between the two binding sites (Fig. 10). Adsorption on the gold surface leads to a narrowing of this distribution compared to simulations in water, indicating a stabilizing effect of the adsorption. Furthermore, adsorption of both domains leads to a relative orientation of the two binding sites only rarely observed in free simulations. The observed binding-site reorientation raises interesting questions about the physiological activity of surface adsorbed multi-domain proteins, even if the individual fold of each domain may structurally not be affected by adsorption. For the here simulated domains FN9–10, it has been suggested that the structural stability of FN9–10 is important for the proper ligation of the protein with the cell-surface receptor integrin A5B1 [38], and that the relative orientation plays an important role in mechanosensing [39], [40]. Hence, surface adsorption of adhesion molecules might potentially alter their signaling properties, as already suggested earlier [41]. Further, these reorientations can expose hidden epitopes, potentially causing an immunological threat.

### Impact of Mutations

Since our results of both the model peptide and the protein imply an important role for *Arg* in early steps of the adsorption process, we mutated all *Arg* in silico to *Val* or to *Lys*. The *Arg*→*Lys* mutation retains the charge distribution on the FN surface, while the *Arg*→*Val* mutation leads to an overall highly negatively charged protein. For both mutants, we performed 100 ns adsorption simulation each. Our results underline the importance of electrostatic properties for the adsorption: while the *Arg*→*Lys* mutant adsorbs very similar to the WT, the *Arg*→*Val* mutant needs longer time for adsorption, only adsorbs at the rather uncharged ends of the protein and bridges the two gold layers. Hence, the desolvation penalty of the negatively charged protein is too high to allow fast and strong adsorption. This has strong implications for the design of specifically adsorbing proteins: it is very desirable to create proteins that adsorb in a specific orientation onto the gold surface. For such an effort, comparable to drug design or to the design of protein-protein interaction, two aspects need to be regarded. First, one has to create a surface patch that easily adsorbs onto the surface (design-in). But second and equally important, one has to design the remaining of the surface such that it is unlikely to adsorb (design-out). Our results give first hints for both strategies: overall neutral patches containing *Arg* are preferred binding sites (desgn-in). Negatively charged areas hinder adsorption (design-out).

### Summary

Our results of the two weakly related systems, which mainly share a common secondary structure, reveal certain similarities, which may be regarded as common topics in protein adsorption on gold: the adsorption in both cases is a step-wise and cooperative process. When closely analyzing the adsorption both for the model peptide and the protein, it becomes apparent that *Arg* plays a crucial role. *Arg* residues are either providing the first contact or involved within the following ns after the first contact with the surface (Table 1). No other amino acid has the same behavior. The availability on the protein surface, the high affinity for gold [17], [42] and its length predestine *Arg* as an early anchor for protein-gold interactions. Biotechnological applications may not only require surface-specific interactions, but additionally preferred orientations of proteins on the surface, best without sophisticated covalent attachment schemes. Our simulations indicate that intelligent placements of *Arg* could fulfill such a role. Furthermore, β-sheet rich proteins are stable on the gold surface, but domains may re-orient impacting biological effects.

**Table 1:** For each simulation and residue, the first contact time of a specific residue type is given.

AA/Simulation	0°	60°	120°	180°	240°	300°	N-Term	C-Term	Average first contact time
ACE	***0.275***	1.45	20.2	42.375			0.0		12.86
ALA		2.275	20.8	37.45		84.4	9.975	28.825	30.62
ARG	0.75	***0.025***	0.1	***0.475***	***0.05***	0.9	***0.025***	***0.175***	***0.31***
ASN	30.7			18.55	5.425	0.3		52.95	21.59
ASP	59.275	4.875	1.65	4.7	0.175	0.05	20.775		13.07
GLN	23.95	21.725	17.575	36.6					24.96
GLU	1.15	11.525	60.4	43.25	4.6	18.875	22.775	43.525	25.76
GLY	2.475	1.225	1.65	5.725	4.55	0.85	10.1	0.2	3.35
HIS	0.025	21.625	60.425	4.075	4.625	46.65			22.90
ILE	5.8	21.325	***0.075***	36.9				58.25	24.47
LEU	5.45	7.975	20.75			21.975	20.775		15.39
LYS			7.15	2.6					4.88
NAC	54.25	1.225						0.4	18.63
PHE	0.875	71.2	73.975	31.375	81.7	41.6			50.12
PRO	2.875	1.75	60.5	0.7	4.625	***0.05***		0.0	10.07
SER	1.55	30.55	2.1	0.025	6.8	0.125			6.86
THR	30.75	1.95	1.65	1.15	4.575	22.025	0.1	***0.175***	7.80
TYR		2.05		4.4				28.9	11.78
VAL	19.975	11.3	20.525	40.9	69.875	60.9		19.275	34.68

Criterium for a contact is a distance Δz from the surface <0.3 nm, as measured from the atom closest to the surface. Time unit is ns. The last columns provide averages over the first contact times in the simulations. Caps are listed as ACE (acetyl) and NAC (n-acetyl). The lowest time for each column is highlighted in bold (for N-Term and C-Term simulations, contact times of ACE and NAC, respectively, are 0.0 ns by construction). *Arg* covers ∼13% from the total Solvent Accessible Surface (SAS) of the studied portion of Fibronectin (Domain 9 & 10).

### Conclusions

We have presented long atomistic molecular dynamics simulations of complex polypeptides on a polarizable gold(111) surface in water. On the time scale of the simulation, no unfolding has been observed. However, we did observe significant domain-domain re-orientation, which may promote the exposure of cryptic epitopes [41]. The simulations have also demonstrated the crucial role of *Arg* in promoting early on in the adhesion process a direct contact with the gold surface, similar to what has recently been shown for the adsorption of Lysozyme on a charged model surface [43]. Leveraging these special properties of *Arg* may help to design proteins adsorbing on a gold surface in a specific orientation without the need for sophisticated covalent coupling schemes. Taking into account that *Arg* is cationic at neutral pH, our result hint at the possibility to electrochemically control the protein-gold interactions, since the gold surface can be made positively charged (and thus *Arg*-repelling) by an applied potential bias. Hence, our simulations offer a first in-depth view into the possibilities to rationally control the binding of proteins on gold.

## Methods

We used fibronectin domains FN9–10 from crystal structure 1FNF [44] as input (starting from residue 1326). The structure was placed in 6 rotations in 60° steps parallel and 2 conformations perpendicular to the gold surface with a water layer separating the protein from the gold surface (Fig. 4). In the solvated box, Na and Cl ions were placed in order to obtain physiological conditions of 150 mMol and an overall neutral box. The obtained systems were energy minimized and then subject to 500 ps relaxation with restraints on all heavy atoms followed by a separate 50 ps simulation with semi-isotropic Parrinello-Rahman pressure coupling [45] in z-direction. Compressibility was set to 0 m^2^N^−1^ in xy-direction. The obtained structures were then simulated for 100 ns. All simulations were performed with the Gromacs 4.0.7 package [46], integration time steps of 2 fs and a Nose-Hoover thermostat [47] at 300 K. Particle Mesh Ewald [48] for long range electrostatics above 1.1 nm and switch cutoff 0.9–1.0 nm for van der Waals interactions was used. GolP [13] and OPLS/AA parameters [49] were used for the surface and the protein and the SPC water model were used. For distance and secondary structure analysis, we used the gromacs tools g_traj and do_dssp, which is a trajectory interface to the dssp program [50]. The simulation of RAD was performed by placing the equilibrated RAD β-sheet [31] parallel to the gold surface at a distance that do not prevent the formation of a complete water monolayer between the peptide and the surface. The β-sheet fragment is composed of 16 RAD16II peptide molecules. After minimization, the NVT dynamics was started and run for 100 ns. Values of the adsorption energies and of its components (see Fig. 1) were collected every 0.2 ps during the entire dynamics. From them we subracted the energy averaged over the first ns of the production dynamics (when the peptide is not in contact with the surface) to define the time-dependent adsorption energy reported in Fig. 1. The bonded term (blue) also includes the Lennard-Jones and Coulomb interactions between the peptide atoms separated by three bonds (1–4 interactions), as their parameterization and meaning is entangled with those of the bonding terms. To smooth out statistical fluctuations in the energy profiles, shown in Fig. 1, we present values obtained by averaging the energy on a 1 ns window centered on each collected energy. The use of smaller averaging windows yield similar trends but noiser profiles (see Fig. S1). We also recall that the image charge procedure used here provides correct statistical sampling and free energies [51] but may overestimate the image contribution to the interaction enthalpy. No entropic term is included in Fig. 1.

## Supporting Information

Figure S1 **Impact of averaging of the potential energy of the system.** Potential energy of the system as obtained directly from the dynamics (Potential E) and averaged over time windows of different lengths.
(TIF)
