# Supplementary figures and images for: Interaction of β-Sheet Folds with a Gold Surface
Source: PLoS One. 2011 Jun 7;6(6):e20925. doi: 10.1371/journal.pone.0020925 (PMC3110247; doi:10.1371/journal.pone.0020925)

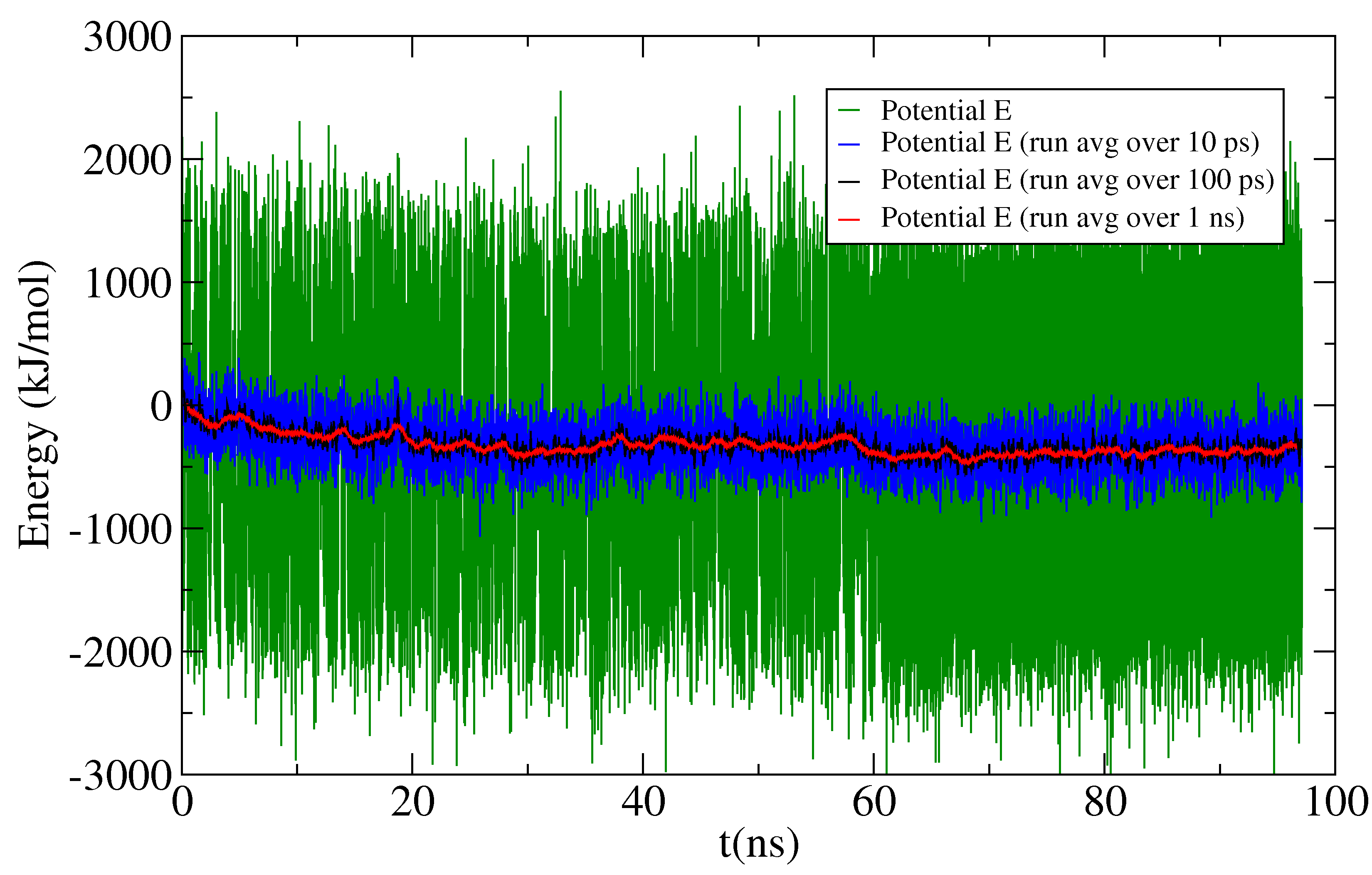

Supplement: Figure S1 — Impact of averaging of the potential energy of the system. Potential energy of the system as obtained directly from the dynamics (Potential E) and averaged over time windows of different lengths. (TIF) [file pone.0020925.s001.tif]
